# Translation of Trosseau and Bellog, on Laryngitis

**Published:** 1840-01

**Authors:** 


TRANSLATION OF TROSSEAU AND BELLOG ON LARYNGITIS.

We are happy to perceive that one of our Collaborators, Dr. Warder, of Cincinnati, has translated and published in the valuable "Select American Library," of Professor Dunglison, the interesting work of Trousseau and Bellog on Laryngitis; which we beg leave to commend to our readers, as containing much new and important information, on an increasing variety of pulmomary disease. The readers of the *Western Journal of the Medical and Physical Sciences*, will recollect the instructive analytical review of that work, made by Dr. Warder, and published in the 11th volume. As far as we recollect, his translation of this volume from the French, is the first which has been made of any extended work, by a Western physician. D.

